# Southern Medical College—Opening Exercises

**Published:** 1886-10-20

**Authors:** 


### SOUTHERN MEDICAL COLLEGE.—OPENING EXERCISES.

The following account of the opening exercises of the Southern Medical College, on the 6th instant, is copied from the Evening Journal of that date :

The Eighth Annual Course of Lectures in the Southern Medical College was inaugurated under most favorable auspices on yesterday at 11 a. m., in the spacious college edifice on Porter street. A larger number of students than had ever been present at the opening exercises filled the corridors in advance of the hour, and at the sound of the bell flocked into the lecture room, where a great many ladies and gentlemen were already assembled. Groups of interested people of the better class of our refined population continued to arrive until the entire sitting capacity of the commodious hall was occupied, and a large crowd of gentlemen were compelled to remain standing at the rear.

After a little delay, caused by the throng of visitors coming in at the moment, Dr. T. S. POWELL, the President of the College, arose and announced that an address would be delivered by a speaker so well known that his name need not be given, and presented Dr. G. G. ROY, Professor of Materia Madica and Therapeutics, to the vast assembly, amid the cheers of the men and the smiles of the ladies.

Upon appearing before the audience Dr. ROY seemed to be inspired by the gratifying reception extended to him. With a radiant countenance he entered upon his work, and delivered the opening address. After a chaste and appropriate peroration upon the faithful performance of duty in all the departments of life, as the factor of success, he announced as his subject : "Some of the Essential Duties of the Doctor to Himself and to his Patients." In treating his subject, he discussed the duties of the doctor to himself in their physical, intellectual and moral aspects. He announced as the doctor's first duty to himself, in all the relations of life, was to be a Gentleman in the strictest sense of this pure English word. He defined what Webster, Hare, and Locke considered the requisites of a gentleman, and urged the students to make these their standard. He counseled neatness of person and attire, with kindness, gentleness, and sympathy, especially for those in the humble walks of life. He urged as a special duty of the doctor to himself, to preserve by all legitimate means a *mens sana in sans corpore.* He showed the greater liability of the doctor to most of the diseases common to the human race, and quoted statistics from Cuspar, Escherchs, and Ogles, showing that three-fourths of the doctors die before they reach fifty years, and that the life of the doctor is shorter than that of the lawyer, teacher, preacher, merchant or artist. He brought out two significant facts from these statistics, in the large per cent. of doctors who die from alcoholism and who commit suicide ; and from the first of these facts he took occasion to give the views of Dr. Benjamin Rush, uttered one hundred years ago, and more recently of Dr. Richardson, of London, and of Sir Henry Thompson, upon the use of alcoholic stimulants either for dietetic or medicinal purposes, as doing far more harm than good. In fact, these great men teach that they are not necessary at all for the promotion of health or the cure of disease. He urged the Class to practice Temperance and teach it by example and precept. After pointing out other wholesome lessons of duty of the doctor to himself, he discussed the second branch of his subject—*i. e.* the duty of the doctor to his patients. He said honesty in dealing with his patients and their families was one of the first and foremost duties of the doctor. Never deceive the family or friends of your patients as to their true condition. It is a bounden duty to tell them the truth at all times when an opinion is asked. The doctor should endeavor to arrange his professional labors so as to do as little work as possible on the Sabbath. The doctor should lay up a full storehouse of medical knowledge, by constant and earnest study and by the fruitful lessons of experience. The doctor must be prompt in filling all professional calls or appointments ; never promise to visit a patient in an hour and put it off till to-morrow He begged that none of the graduates of the Southern Medical College should degenerate into Quack Medicine-makers or vendors, thus prostituting their diplomas to illegitimate ends, and bringing shame and disgrace upon their Alma Mater. He urged the students to be faithful to every trust—to unite with every worthy member of the grand profession to build and keep in repair the magnificent temple of the science and art of Medicine and Surgery, and let their lives be so illustrious in good deeds that humanity, God and eternity will be blest by their having lived.

At the conclusion of Dr. ROY's address, Dr. J. McF. GASTON arose from his seat in the auditory and made the following remarks, while Dr. POWELL remained standing at the desk within the railing :

*Mr. President:* Allow me to interrupt your course of proceedings with a few words. Trusting that this casket which I hold in my hand may not prove a Pandora's box, nor that the bearer may be feared as was the Greeks of old who came bearing presents, no apprehension should be felt by you. I would say to the ladies and gentlemen in attendance that my predecessor in the chair of Surgery, in retiring from the Southern Medical College, carried with him such pleasing recollections of his associations with our worthy President that he has sent back a kind memento of personal and professional consideration for him during their relations in this Institution. Dr. JOHN THAD. JOHNSON found a nugget of gold in that region to which his steps were directed, that was sent to our Medical Faculty with the injunction to have it fashioned in a form to serve as a souvenir of his esteem for President THOMAS S. POWELL, and that it should be presented to him.

I am pleased to inform you, Dr. POWELL, that it devolves upon me to represent the Faculty in conveying this evidence of high appreciation by our former colleague, whose heart turns to you with the affection of a son for his father. This testimonial has been prepared in the most approved style of Art, with the expressive motto, *Virtus in arduis*, indicating that energetic persistence in the performance of duty which has always characterized your undertakings, and which has always been crowned with such eminent success. Among the trophies of your successful career stand prominently the commodious College building in which we are assembled to-day and the growing prosperity of this Institution, which was inaugurated here six years ago. The present Faculty have much satisfaction in indorsing this testimony of their former colleague to your zealous interest for a high standard of Medical Education in founding the Southern Medical College. The progressive improvement in the course of instruction, with the provisions for illustration in all its departments, bespeaks for this undertaking a favorable consideration by all who are interested in the advancement of the medical profession. Through your influence the Ladies of Atlanta have contributed greatly to the relief of the sick by establishing the *Ivy Street Hospital*, and their efforts are now directed to enlarging and extending the facilities for treating all classes of patients. Persons who are seeking objects of beneficence may endow Scholarships in the College and Beds in the Hospital, or make donations for the original investigations and the advancement of Science and it would prove the crowning glory of your life to promote these noble objects. It is confidently expected that men and women, with generous impulses and ample means, may be found ready to contribute for the success of these laudable undertakings. Those qualities of head and heart indicated by *Virtus in arduis* have been worthily exemplified by your past achievements, and we trust, as you go forward and upward in higher and greater attainments, that *Crescit eundo* may be appropriately inscribed upon this testimonial to your fair fame and good name, which is now delivered to you to be worn as a badge of honorable distinction.

This beautiful testimonial, fashioned in the most chaste style, being handed to Dr. POWELL, he responded under evident emotion, indicating that he was most profoundly impressed by the reception of this evidence of cordial regard from his friend and former colleague, whose home is now at Ætna, Cal.

Among other important remarks, President POWELL presented the following striking points in his happy reply: It has often been surprising to me that a thorough pessimist could find birth and sustain existence in a world like this of ours. Despite the dark sides of this life, it has so many bright and tender tones of coloring, we often catch glimses and inspirations of Eden-like beauty that cheer and buoy up our spirits amid the darkest gloom, and make our hearts open to catch the refreshing dews of man's love to man, as a flower unfolds its petals to the cool kisses of a Summer night. These thoughts passed through my mind while my distinguished friend and colleague, Dr. GASTON, was so kindly addressing me just now, and then, to crown it all, presented to me this highly prized souvenir from my noble friend and former colleague who is now making his home on the golden shores of California. It is needless to say, my dear doctor, that the presentation of this memento of artistic and beautiful workmanship by Dr. THAD JOHNSON, and the Faculty of this Institution, is to me a glimpse of one of the brightest and most refreshing incidents I could meet with in the later years of my professional life. Its memory will be to me grateful and everlasting, and this medal, the eloquent token of fraternal affection and appreciation, shall be treasured as one of my most valued possessions. Of the flattering estimate expressed to-day of my personal ability, shown in my career, I know not how to thank those noble hearted professional friends from whom it comes, especially as I so deeply feel how little I have done commensurate with what I trust was a worthy ambition. It never has been difficult for me to have faith in a good cause. If an enterprise has the approval of Heaven, of noble men and women, and is pushed forward with zeal, energy, perseverence, and all legitimate methods, I can see no such word as Fail written in its undertaking. If I know I am right, I never fear obstacles. If they are small and insignificant they are the more easily removed ; if they are large, it only requires greater and grander efforts to overcome them.

Rev. Sam Jones has said that he would not mind being swallowed by a whale, but he hated to be nibbled by minnows. I think the Rev. Sam is right ; but we must remember he has not much flesh to spare ! Now, if I had to undergo either ordeal, unless I should first lose some of my present avoirdupois, I think it would require considerable of a whale to take me in ! If the minnows should tackle me, I would only tell them to nibble on, as there was enough for a whole school of their sardine tribe !

As to the generous confidence given me by my friends and colleagues, I can only show my gratitude by striving to be still more worthy of their appreciation, and repeat here that in every work for individual or public good, especially for the Youth of our land, my heart and my hands are ready to be engaged.

I am proud of the motto engraved on this medal, and I would especially urge the young men present, and more especially those who have chosen the medical profession, to take "*Virtus in arduis*" for a watchword, and to remember that, under God, "All things come to those who watch and wait."

And now, gentlemen of the Faculty, to you, and to my good friend Dr. THAD. JOHNSON, I have not words to express my feelings in regard to the honor you have done me on this occasion. I can only lay my hand on my heart, full of grateful emotions, and make you a respectful obeisance.

